# The microbiota–metabolite–immune axis in colorectal cancer: mechanistic insights and emerging clinical applications

**DOI:** 10.3389/fimmu.2026.1768792

**Published:** 2026-05-28

**Authors:** Bin Sun, Tong Wang

**Affiliations:** 1 The Affiliated Wuxi People’s Hospital of Nanjing Medical University, Wuxi, China; 2 Wuxi Medical Center, Nanjing Medical University, Wuxi, China; 3 Wuxi People’s Hospital, Wuxi, China

**Keywords:** colorectal cancer, gut microbiota, inflammation, microbial metabolites, tumor microenvironment

## Abstract

Colorectal cancer (CRC) results from a complex interplay of host genetics, environmental factors, and gut microbiota. Increasing evidence suggests that intestinal microorganisms significantly affect the initiation and progression of CRC through metabolic and immunological reprogramming. Dysbiosis, defined as an imbalance between beneficial and harmful microbial species, leads to chronic inflammation, genotoxic stress, and disruption of epithelial homeostasis. Microbial metabolites, such as short-chain fatty acids, secondary bile acids, and tryptophan derivatives, function as signaling molecules that influence epithelial proliferation, apoptosis, and immune cell activity. These metabolites regulate essential oncogenic and inflammatory pathways, including Wnt/β-catenin, NF-κB, and STAT3, and alter the tumor microenvironment by affecting regulatory T cells (Tregs), Th17 cells, macrophages, and myeloid-derived suppressor cells. Specific bacteria, such as *Fusobacterium nucleatum*, *enterotoxigenic Bacteroides fragilis*, and colibactin-producing *Escherichia coli*, illustrate how particular microbes can promote tumorigenesis through metabolite-mediated signaling and immune modulation. This review summarizes recent advances in understanding how gut microbiota and their metabolites contribute to colorectal carcinogenesis by influencing inflammatory signaling, epithelial homeostasis, and tumor immune responses. These mechanistic insights highlight the microbiota–metabolite–immune axis as a crucial driver of CRC initiation and progression. The increasing recognition that microbial alterations occur alongside early neoplastic changes and affect tumor behavior emphasizes their translational potential. Although further validation in large, well-controlled clinical settings is necessary, microbiome- and metabolite-based markers could enhance current strategies for the early detection, risk assessment, and therapeutic guidance of CRC. Ultimately, deepening our understanding of the intricate interactions between intestinal microbes, host metabolism, and immune regulation will facilitate the development of microbiome-informed approaches for CRC monitoring and intervention in the future.

## 1 Introduction

Colorectal cancer (CRC) continues to pose a significant global health challenge, with around 1.9 million new cases and 900,000 deaths reported annually (1). Genetic mutations in tumor suppressor genes (e.g., APC, TP53) and oncogenes (e.g., KRAS, PIK3CA) are central to the pathogenesis of CRC. However, environmental factors, especially the gut microbiota, have also emerged as significant determinants in tumor initiation, progression, and therapeutic response (2, 3). The gut microbiota, made up of trillions of bacteria, viruses, fungi, and archaea, plays a crucial role in maintaining intestinal homeostasis. It achieves this by regulating nutrient metabolism, supporting epithelial barrier function, and modulating immune responses (4). Dysbiosis—defined as a shift toward pathogenic taxa or reduced microbial diversity—has increasingly been recognized as a hallmark of CRC (5, 6). Multiple studies have shown significant changes in the gut microbial composition of CRC patients (7). Procarcinogenic bacteria such as *Fusobacterium nucleatum*, *Escherichia coli*, and *Peptostreptococcus* spp. are consistently enriched in fecal and tumor samples, whereas beneficial commensals including *Akkermansia muciniphila*, *Faecalibacterium*, and *Bifidobacterium* are markedly depleted (8, 9). Among these, *A. muciniphila*—a symbiont that resides in the intestinal mucus layer—has garnered significant attention due to its ability to enhance mucosal barrier integrity, modulate immune responses, and improve metabolic homeostasis. These characteristics make it a promising candidate for probiotic use (10).

Beyond microbial composition, bacterial metabolites have emerged as crucial mediators connecting dysbiosis to colorectal tumorigenesis. Among these metabolites, bile acids are one of the most influential classes derived from microbiota, playing essential roles in maintaining intestinal and immune homeostasis (11). Microbial conversion of primary to secondary bile acids modulates chemokine expression, immune cell recruitment, and antitumor immunity along the gut–liver axis (12). While the excessive accumulation of certain secondary bile acids promotes intestinal inflammation and carcinogenesis, others, such as ursodeoxycholic acid, have protective effects. These beneficial bile acids can help mitigate inflammatory bowel disease and reduce the recurrence of adenomas (13). These findings highlight the complex interactions between microbial composition, metabolite production, and immune regulation. They suggest that targeted modulation of the gut microbiota could be a promising strategy for the prevention and therapy of CRC (7).

Understanding the microbiota–metabolite–immune axis offers a comprehensive framework for connecting the microbial, metabolic, and immunological mechanisms involved in colorectal cancer (CRC). This review focuses on three key areas: (i) the role of gut microbiota dysbiosis in contributing to CRC; (ii) the dual role of microbial metabolites as either oncogenic or protective mediators; and (iii) the microbiota-mediated immune and signaling mechanisms that influence colorectal cancer.

## 2 Gut microbiota dysbiosis in colorectal cancer

Patients with CRC consistently exhibit alterations in gut microbiota composition when compared to healthy individuals. CRC-associated dysbiosis is typically characterized by the enrichment of pathogenic bacteria—such as *Fusobacterium nucleatum* (*F. nucleatum*), enterotoxigenic *Bacteroides fragilis* (ETBF), and colibactin-producing *Escherichia coli* (CoPEC)—accompanied by a depletion of beneficial commensals including *Faecalibacterium prausnitzii* (*F. prausnitzii*), *Lactobacillus*, and *Bifidobacterium* species (**Table 1**). These compositional shifts collectively contribute to mucosal inflammation, genomic instability, and dysfunction of the epithelial barrier, which in turn drive colorectal tumorigenesis (14).

**Table 1:** Special bacterium related to colorectal cancer (CRC).

Microorganism	Expression/role in affecting CRC	Function
*F. nucleatum*	Driver	Enriched in CRC; Adhesion and Epithelial Disruption (18); Inflammation and Immune Evasion (19, 20); Metabolic Reprogramming (24)
*Escherichia coli*	Driver	DNA alkylation–induced genomic instability (25–27); Senescence-associated inflammatory signaling (28); EMT and stemness induction (29); Immune suppression (30); Metabolic reprogramming (31)
*Enterotoxigenic Bacteroides fragilis*	Driver	Intestinal barrier disruption (32); Th17-driven inflammation (33, 34); Epigenetic and immune remodeling (35, 36)
*F. prausnitzii*	Protector	Anti-inflammatory and immune modulation (37, 38); SCFA-mediated metabolic regulation (38, 39)
*Bifidobacterium*	Protector	Enhancement of barrier integrity (40); Suppression of inflammatory cytokines (41); Remodeling of gut microbiota (42)
*Lactobacillus*	Protector	Enhanced dendritic cell infiltration in the tumor microenvironment (43); Chenodeoxycholic acid–induced mitochondrial dysfunction and oxidative stress–mediated apoptosis (44); Induced apoptosis and inhibited CRC cell growth (45)
*Clostridium septicum*	↑	Associated with poor prognosis and advanced disease (46)
*Eubacterium rectale*	Driver	Activate NF-κB signaling to induce proinflammatory immune responses (47)
*Helicobacter pylori*	Driver	Associated with increased CRC incidence and mortality (48); Driven immune dysregulation (49);
*Streptococcus bovis*	↑	Activation of the Wnt pathway (50);

Driver, microorganism that promotes CRC development; Protector, microorganism that suppresses CRC development; ↑, increased abundance in CRC patients compared to healthy controls.

### 2.1 F. nucleatum

*F. nucleatum* has become one of the most extensively researched bacteria associated with CRC. Typically a commensal organism found in the oral cavity, its levels are significantly increased in both the feces and tumor tissues of CRC patients. This heightened abundance correlates with more advanced disease stages and poorer prognoses (15, 16). Genomic studies have revealed distinct *F. nucleatum* subspecies with varying oncogenic potential. Among these, the *animalis* subspecies clade C2 (Fna C2) is highly enriched in CRC and harbors an expanded repertoire of virulence genes—including *fadA*, *fap2*, *cmpA*, and *fusolisin*—that confer enhanced colonization and biofilm-forming capabilities (17).

**Figure 1:**
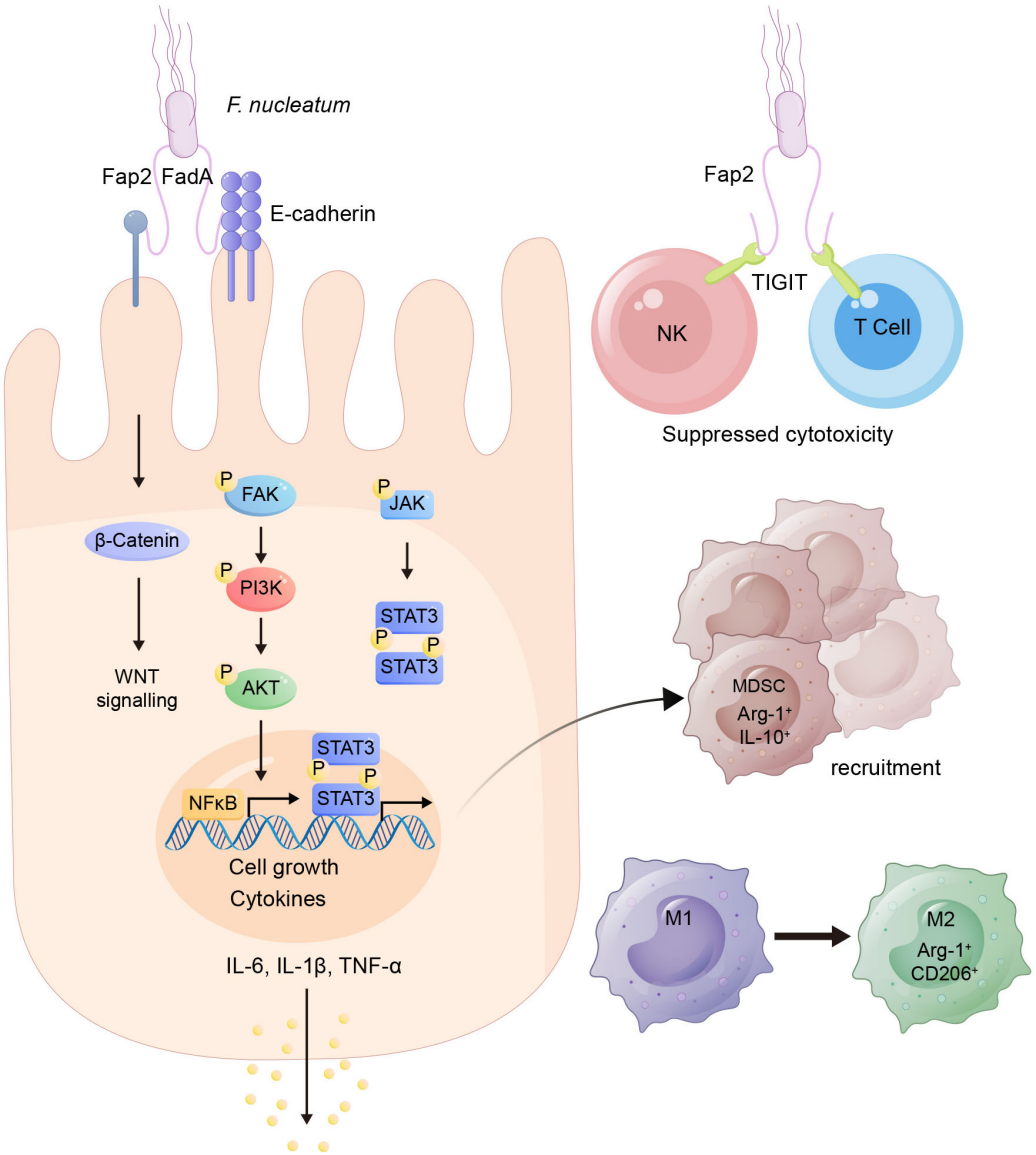
Mechanistic model of *F. nucleatum*–driven colorectal tumorigenesis. *F. nucleatum* promotes colorectal carcinogenesis through multiple synergistic mechanisms. (1) The adhesin FadA binds to E-cadherin on colonic epithelial cells, disrupting intercellular junctions. This disruption activates β-catenin signaling, which promotes epithelial proliferation and initiates tumor formation. (2) The outer membrane protein Fap2 interacts with the inhibitory receptor TIGIT on natural killer (NK) cells and cytotoxic CD8^+^ T cells, suppressing antitumor immunity and facilitating immune evasion. (3) In parallel, *F. nucleatum* induces the secretion of proinflammatory cytokines (IL-6, IL-1β, TNF-α) and activates NF-κB and STAT3 signaling pathways, leading to recruitment of MDSCs and polarization of macrophages toward an M2-like immunosuppressive phenotype, thereby establishing a tumor-promoting microenvironment.

Mechanistically, *F. nucleatum* promotes CRC progression through multiple synergistic pathways. The adhesin FadA binds to E-cadherin on epithelial cells, which disrupts intercellular junctions and activates β-catenin signaling. This process stimulates epithelial proliferation (18). The outer membrane protein Fap2 engages TIGIT on NK and T cells, suppressing cytotoxic activity and facilitating immune evasion (18). In addition, *F. nucleatum* stimulates the secretion of proinflammatory cytokines including IL-6, IL-1β, and TNF-α. This activation of NF-κB and STAT3 signaling pathways further promotes the recruitment of myeloid-derived suppressor cells (MDSCs) and drives the polarization of macrophages toward an M2 phenotype (19) (**Figure 1**).

Beyond its effects on the immune system and epithelial cells, *F. nucleatum* also reprograms tumor metabolism. Recent metabolomic analyzes show that infection leads to enhanced lipid accumulation, disruption of bile acid metabolism, and upregulation of lipid metabolic enzymes, including fatty acid synthase (FASN) and carnitine palmitoyltransferase 1 (CPT1). These changes ultimately promote tumor growth and contribute to chemoresistance (20). Collectively, these findings underscore *F. nucleatum* as a multifaceted driver of CRC that orchestrates epithelial activation, immune suppression, and metabolic reprogramming.

### 2.2 Colibactin-producing *Escherichia coli*

Among CRC-associated bacteria, CoPEC represents a direct microbial source of genomic instability (21, 22). Certain *E. coli* strains harbor the *pks* genomic island, encoding a hybrid polyketide–nonribosomal peptide synthase complex responsible for synthesizing colibactin, a genotoxic secondary metabolite (22). Colibactin alkylates host DNA, leading to the formation of interstrand cross-links and double-strand breaks. This process activates DNA damage response pathways, promotes mutagenesis, and contributes to chromosomal instability (23, 25, 26). Chronic colonization with CoPEC exacerbates tumor development in colitis-associated cancer models (AOM/DSS) (28). Colibactin-induced genotoxic stress mechanistically triggers a senescence-associated secretory phenotype (SASP) through p53 SUMOylation and miR-20a-5p/SENP1–mediated regulation. This process results in the production of hepatocyte growth factor (HGF), which supports tumor growth and the proliferation of neighboring uninfected cells (51). Large-scale genomic analyzes have identified a unique alkylation-associated mutational signature linked to colibactin exposure in human CRC genomes. This finding establishes a direct bacterial mutational imprint (25). Recent metabolomic and spatial microbiome studies have revealed a metabolic dimension to CoPEC-driven carcinogenesis (31). In right-sided CRC, CoPEC is found within tumor niches that are rich in glycerophospholipid metabolism and lipid droplet accumulation. This localization correlates with decreased CD8^+^ T-cell infiltration and immune suppression. The lipid remodeling driven by colibactin, facilitated through the Land’s cycle, enhances the turnover of phosphatidylcholine, providing energy advantages and contributing to chemoresistance. Interestingly, pharmacological inhibition of acyl-CoA synthetase can restore drug sensitivity, indicating a potential therapeutic vulnerability in tumors colonized by CoPEC.

Furthermore, anti-TNF therapy reduces CoPEC-driven CRC progression in murine models by reshaping the microbiota and decreasing inflammation. However, this effect diminishes with cohousing or microbiota transfer, highlighting the significant role of microbial plasticity in this process (30). In tumor epithelial cells, colibactin exposure not only induces DNA damage and G2 arrest but also promotes epithelial-to-mesenchymal transition (EMT), cancer stemness, and immune evasion characterized by diminished CD3^+^ and CD8^+^ T-cell infiltration (29). Additionally, *E. coli*–derived cytotoxic necrotizing factor 1 (CNF1) induces oxidative DNA damage and chromosomal instability through Rho GTPase activation, leading to dysplastic crypt foci and adenoma formation in inflammation-prone mice (27). These findings position CoPEC as a powerful microbial mutagen and a multifaceted oncogenic driver. By inducing DNA alkylation and resulting genomic instability, CoPEC triggers senescence-associated inflammatory signaling and promotes EMT and stemness. Additionally, it facilitates immune suppression and metabolic reprogramming. Together, these mechanisms create a tumor-permissive microenvironment that supports the initiation, progression, and therapeutic resistance of CRC.

### 2.3 Enterotoxigenic *Bacteroides fragilis*

**Figure 2:**
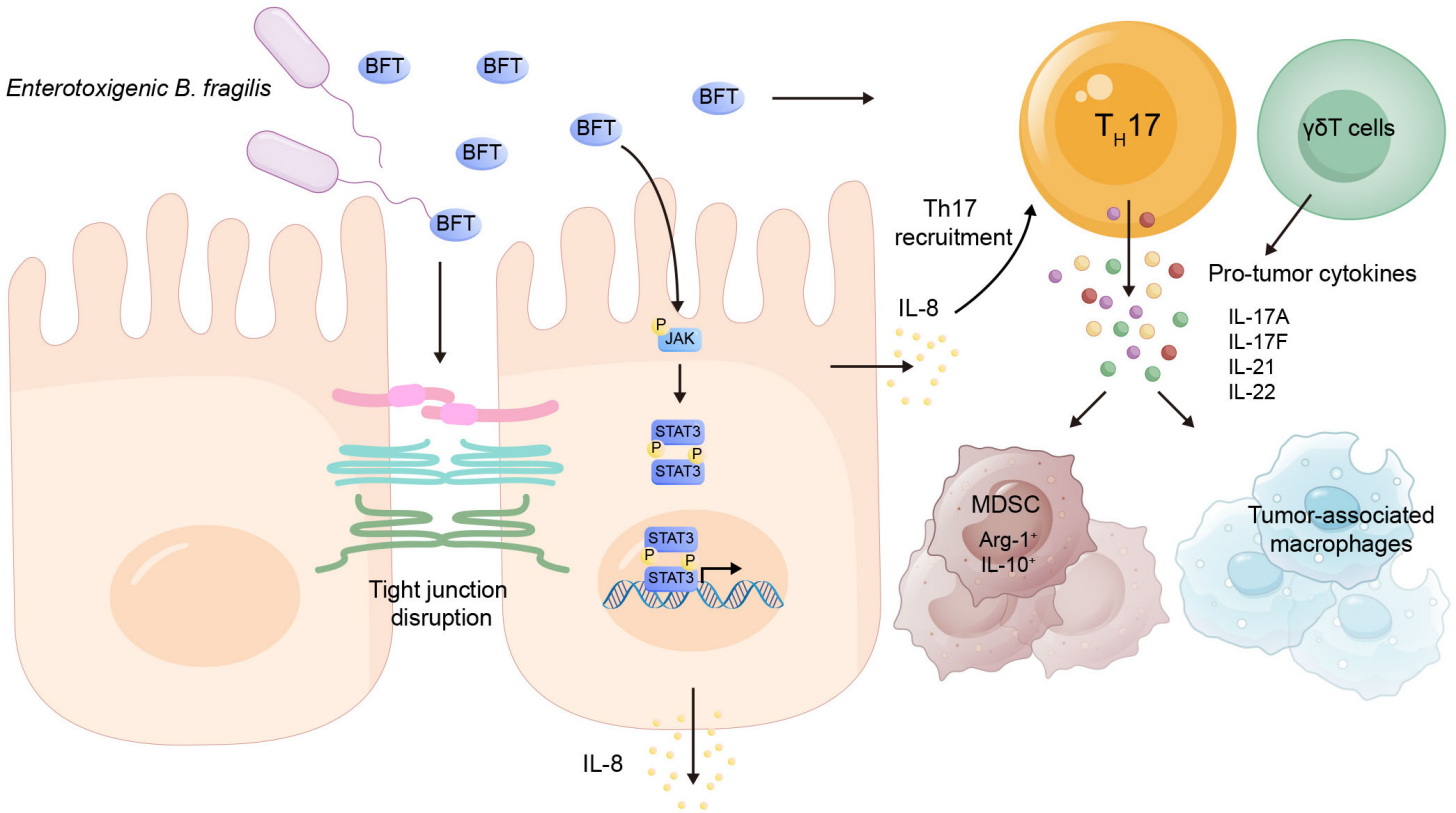
Mechanisms of ETBF-induced colorectal tumorigenesis. ETBF promotes colorectal tumorigenesis via its virulence factor BFT, which disrupts epithelial junctions, activates STAT3-dependent IL-8 secretion, and triggers Th17-driven inflammation. This inflammatory environment reprograms the myeloid compartment, promoting a tumor-supportive phenotype. As a result, there is an expansion of monocytic MDSCs and tumor-associated macrophages, which is dependent on BFT and IL-17.

ETBF promotes colorectal carcinogenesis mainly through its virulence factor, *Bacteroides fragilis* toxin (BFT), which disrupts epithelial tight junctions and induces pro-tumor inflammation (56). The prevalence of ETBF and the *bft* gene is significantly higher in stool and tumor samples from CRC patients, particularly at advanced stages (57–59). Mechanistically, ETBF activates the STAT3 pathway and drives a robust colonic Th17 immune response—both critical for inflammation-associated tumorigenesis (33). In *ApcMin/+* mice, ETBF (but not non-toxigenic *B. fragilis*) induces colitis and accelerates tumor formation via an IL-17–dependent axis. Neutralization of IL-17 or IL-23R significantly decreases tumor burden, thereby confirming the critical role of the STAT3–Th17–IL-17 cascade in ETBF-driven carcinogenesis (33, 56) (**Figure 2**).

Further studies indicate that ETBF infection alters the colonic myeloid compartment, promoting a tumorigenic phenotype. This change is characterized by the expansion of monocytic MDSCs and tumor-associated macrophages, driven by BFT–IL-17–dependent signaling (34). In parallel, ETBF-induced epithelial barrier disruption is accompanied by downregulation of MUC2, Occludin, and ZO-1; induction of EMT through STAT3/ZEB2 signaling (32), and promotion of cancer stemness via JMJD2B-mediated NANOG activation under TLR4–NFAT5 regulation (35). ETBF also enhances IL-8 secretion through STAT3 activation, perpetuating chronic inflammation and tumor progression (36). ETBF contributes to CRC by coordinating barrier disruption, driving Th17 inflammation, reprogramming myeloid cells, and modulating epigenetic factors.

### 2.4 F. prausnitzii

*F. prausnitzii* is one of the most abundant commensal bacteria in the healthy human gut and plays a significant role in producing the short-chain fatty acid (SCFA) butyrate. It is widely acknowledged for its essential role in maintaining intestinal homeostasis, acting as a key anti-inflammatory and barrier-protective bacterium (60). Multiple studies consistently report a significant depletion of *F. prausnitzii* in patients with CRC and inflammatory bowel disease. This suggests that the loss of *F. prausnitzii* is a hallmark of dysbiosis linked to intestinal inflammation and tumorigenesis (61–63).

Recent work highlights *F. prausnitzii* as a potent tumor-suppressive commensal in CRC (37). Oral supplementation significantly suppresses tumor growth in CRC models and is associated with increased infiltration of CD8^+^ T cells in both the blood and tumor tissues, indicating enhanced antitumor immunity. Metabolomic profiling reveals a positive correlation between *F. prausnitzii* abundance and serum tyrosol levels, which are markedly reduced in CRC patients. The administration of *F. prausnitzii* elevates tyrosol levels in mice. Tyrosol itself has anti-inflammatory and antioxidant effects by inhibiting NF-κB and HIF-1 signaling, which contributes to the suppression of tumor growth and oxidative stress.

The tumor-suppressive activity of *F. prausnitzii* is primarily attributed to its secreted metabolites. Cell-free supernatants inhibit the proliferation of HCT116 CRC cells (64), implicating soluble factors such as butyrate. Reduced SCFA—especially butyrate—levels are strongly associated with increased CRC risk and poor prognosis (39). Remarkably, the natural compound echinacoside demonstrates oral anti-metastatic efficacy by promoting butyrate-producing bacteria, such as *F. prausnitzii*. This promotion leads to the inhibition of the PI3K/AKT pathway and the reversal of EMT (38). Butyrate synthase inhibition abolishes these effects, confirming the functional role of microbial butyrate production. These findings highlight *F. prausnitzii* as a crucial protective commensal that exerts antitumor effects through immunometabolic and antioxidant mechanisms, with tyrosol and butyrate serving as key mediators. Therefore, targeting *F. prausnitzii* or its metabolites presents a promising strategy for the prevention and therapy of CRC.

### 2.5 Bifidobacterium

*Bifidobacterium* is a key probiotic genus in the human gut, playing essential roles in maintaining intestinal homeostasis, regulating the immune system, and preserving the epithelial barrier. Clinical and experimental studies consistently show that individuals with CRC experience a significant reduction in the abundance of *Bifidobacterium*. This decrease is often associated with lower levels of beneficial metabolites like conjugated linoleic acid (CLA) and butyrate, both of which are inversely correlated with the severity of CRC (65). Functionally, *Bifidobacterium* exerts tumor-suppressive effects through multiple complementary mechanisms. *Bifidobacterium bifidum* supplementation induces apoptosis in colonic epithelial cells, thereby inhibiting tumor growth (66). Oral administration of *B. longum* subsp. *infantis* enhances epithelial barrier integrity by upregulating tight junction proteins, such as Occludin and Claudin-1. This process also suppresses epithelial activation, resulting in reduced permeability and inflammation (40). Moreover, *B. longum* subsp. *infantis* enhances colonic regulatory T cell (Treg) expansion while downregulating pro-inflammatory cytokines (IL-6, IL-1β, TNF-α), thereby dampening tumor-promoting inflammation (41).

Mechanistically, the CLA-producing *B. breve* CCFM683 inhibits CRC progression by increasing intestinal CLA levels, suppressing NF-κB activation, enhancing the expression of tight junction proteins and MUC2, and modulating gut microbiota in a PPARγ-dependent manner (65). Collectively, these findings highlight Bifidobacterium as a key probiotic genus with multifaceted tumor-suppressive effects—spanning epithelial protection, immune modulation, metabolic regulation, and microbiota reshaping—making it a promising candidate for CRC prevention and adjunctive therapy.

## 3 Microbial metabolites as oncogenic or protective mediators

The gut microbiota profoundly influences colorectal tumorigenesis not only through direct interactions with epithelial and immune cells but also via the production of diverse bioactive metabolites that shape the tumor microenvironment (67). These microbial metabolites act as key signaling molecules regulating epithelial homeostasis, immune activation, inflammation, and therapeutic response. The balance between tumor-promoting metabolites—such as kynurenine and trimethylamine N-oxide (TMAO)—and tumor-suppressive metabolites—such as SCFAs and specific secondary bile acids—critically determines disease progression. Perturbations in microbial metabolic networks can amplify oncogenic signaling, impair antitumor immunity, and exacerbate treatment resistance, whereas restoration of beneficial metabolic pathways offers promising strategies for CRC prevention and therapy. Below, we discuss three representative classes of microbial metabolites—SCFAs, bile acids, and TMAO—and their mechanistic roles in CRC.

### 3.1 Short-chain fatty acids

SCFAs are the most abundant microbial metabolites produced from the fermentation of dietary fibers and carbohydrates in the colon. Among these, butyrate has been the focus of extensive research due to its powerful immunomodulatory and antitumor effects. SCFAs serve as crucial mediators of host–microbiota communication, orchestrating both local and systemic immune responses.

Butyrate plays a dual role in antitumor immunity, acting as both a metabolic substrate and an epigenetic regulator. It enhances glycolysis and oxidative phosphorylation in CD8^+^ T cells while promoting acetyl-CoA availability in B cells. This dual action supports immune activation and metabolic fitness (68, 69). In contrast, malignant epithelial cells preferentially utilize glucose (the Warburg effect), leading to intracellular accumulation of butyrate (55). This metabolic asymmetry converts butyrate into a selective histone deacetylase (HDAC) inhibitor in malignant cells, enabling tumor-suppressive transcriptional reprogramming while preserving immune cell function (70).

Through HDAC inhibition and epigenetic remodeling, butyrate modulates multiple immune populations. It promotes macrophage and dendritic cell maturation (71), promotes Treg differentiation (71, 72), and amplifies IL-12 signaling to strengthen CD8^+^ T cell effector functions (73). Recent studies further indicate that butyrate can reverse T-cell dysfunction and promote the generation of highly functional cytotoxic CD8^+^ T cells via TLR5/NFκB signaling, thereby improving responsiveness to immune checkpoint blockade (74).

Beyond HDAC inhibition, recent chromatin-mapping studies have shown that butyrate and propionate can directly generate histone acylation marks, including butyrylation and propionylation, at promoter and enhancer regions associated with genes involved in differentiation, ion transport, and growth control. These modifications increase chromatin accessibility and may explain the divergent proliferative effects of SCFAs on normal versus CRC cells (75). This evidence further supports SCFAs as epigenetic metabolites that reshape host gene expression in a context-dependent manner.

**Figure 3:**
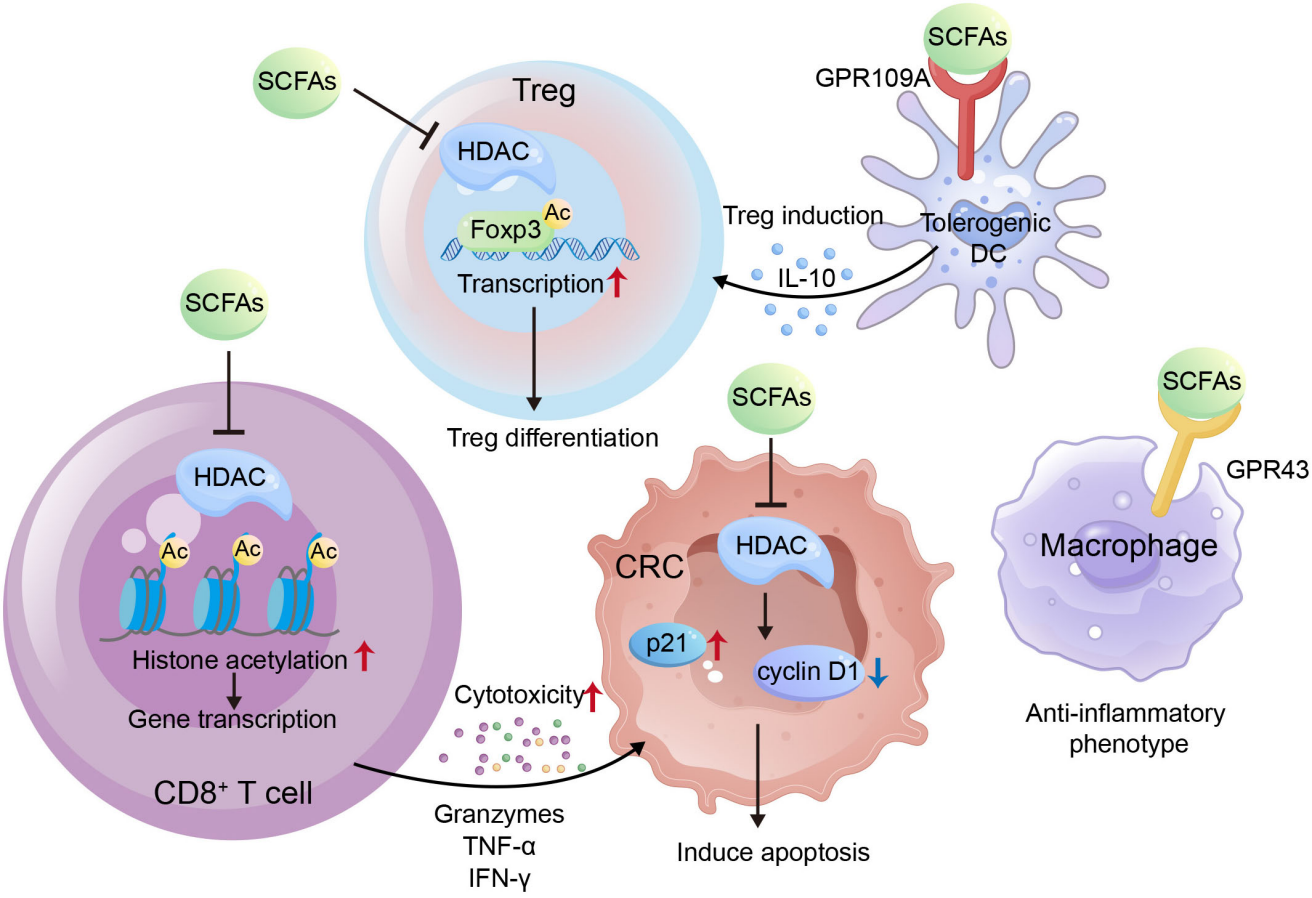
Immunoregulatory and tumor-suppressive functions of butyrate in colorectal cancer. Butyrate-induced apoptosis through GPR109A/GPR43 signaling. GPR109A-mediated induction of Tregs and IL-10^+^ T cells. Butyrate-driven activation of cytotoxic CD8^+^ T cells (80).

Butyrate also signals via G-protein–coupled receptors such as GPR109A and GPR43, which are frequently downregulated in CRC (76, 77). Therefore, restoring their expression enables butyrate to induce apoptosis by suppressing anti-apoptotic proteins (Bcl-2, Bcl-xL, cyclin D1) and activating death receptor pathways (78). GPR109A activation on macrophages and dendritic cells further promotes Treg and IL-10^+^ T cell differentiation, mitigating tumor-promoting inflammation (74). Functionally, butyrate directly inhibits tumor growth by promoting cytotoxic CD8^+^ T cells expressing granzyme B, IFN-γ, and TNF-α (79) (**Figure 3**).

Importantly, specific butyrate-producing bacteria have demonstrated translational relevance in CRC. Roseburia intestinalis, a commensal species depleted in patients with CRC, suppresses tumorigenesis in ApcMin/+ and AOM-induced mouse models by restoring intestinal barrier integrity and increasing butyrate production. In syngeneic CRC models, R. intestinalis or butyrate significantly enhanced anti-PD-1 efficacy by inducing functional CD8^+^ T cells through TLR5/NF-κB signaling (74). *F. nucleatum*–derived butyric acid paradoxically sensitizes microsatellite-stable CRC to anti–PD-1 therapy by inhibiting HDAC3/8 in CD8^+^ T cells, increasing H3K27 acetylation at the Tbx21 promoter and restoring T cell effector function (75). These findings identify beneficial butyrate-producing commensals as potential live biotherapeutic adjuvants for immunotherapy. Paradoxically, even pathogenic bacteria may exploit butyrate-mediated immune activation. Intratumoral Fusobacterium nucleatum has been shown to sensitize microsatellite-stable (MSS) CRC to anti-PD-1 therapy (79). Mechanistically, Fn-derived butyric acid inhibits HDAC3/8 in CD8^+^ T cells, increases H3K27 acetylation at the Tbx21 promoter, represses PD-1 expression through TBX21, and restores T-cell effector function. Clinically, high intratumoral Fn abundance correlates with improved anti-PD-1 responses in MSS CRC, suggesting that microbial metabolic activity may outperform taxonomic classification alone in predicting immunotherapy benefit.

In addition to T-cell regulation, butyrate also remodels the myeloid compartment of the tumor microenvironment. Single-cell transcriptomic analyzes demonstrated that sodium butyrate markedly reduces M2 macrophage polarization and decreases PD-L1^+^ tumor-associated macrophage infiltration through the HDAC/TLR4/MyD88 axis (80). Moreover, butyrate synergized with PD-L1 blockade in preclinical CRC models, indicating that SCFAs may enhance checkpoint inhibitor efficacy by simultaneously reinvigorating lymphoid immunity and dismantling immunosuppressive myeloid niches.

Collectively, these findings identify SCFAs—particularly butyrate—as key mechanistic mediators connecting diet, gut microbial composition, epigenetic regulation, immune checkpoint responsiveness, and tumor microenvironment remodeling in CRC. Through these integrated actions, SCFAs provide a strong rationale for microbiota-based precision strategies that combine dietary modulation, engineered probiotics, metabolite supplementation, and immunotherapy. In addition to butyrate, other SCFAs such as propionate can also reshape chromatin accessibility and enhance the expression of genes involved in cellular growth, differentiation, and ion transport (81). Notably, butyrate exerts context-dependent effects, maintaining epithelial homeostasis in normal intestinal tissue while suppressing proliferation and promoting differentiation in malignant cells. Overall, SCFAs function as critical metabolic and epigenetic interfaces linking microbial activity, intestinal integrity, and antitumor immunity, highlighting their substantial therapeutic promise for the prevention and treatment of CRC.

### 3.2 Secondary bile acids

Bile acids, which are cholesterol-derived metabolites, play essential roles in lipid digestion, microbial ecology, and maintaining intestinal homeostasis. Primary bile acids, such as cholic acid and chenodeoxycholic acid, are synthesized in the liver, conjugated with glycine or taurine, and then secreted into the duodenum. While the majority of these bile acids are reabsorbed in the distal ileum, approximately 5% enters the colon. In the colon, microbial bile salt hydrolases (BSHs) deconjugate these bile acids and transform them into secondary bile acids, such as deoxycholic acid (DCA) and lithocholic acid (LCA), through processes of dehydrogenation and 7α-dehydroxylation (82, 83).

Microbially derived secondary bile acids exert bidirectional effects on CRC. On one hand, 3-oxo-lithocholic acid (3-oxo-LCA) and isoallo-LCA act as immunomodulatory and protective metabolites. 3-oxo-LCA serves as an agonist of farnesoid X receptor (FXR), a master regulator of bile acid homeostasis and intestinal barrier integrity (84–86). FXR expression is frequently downregulated in CRC and inversely correlates with disease progression (87, 88). Activation of FXR by 3-oxo-LCA suppresses the proliferation of intestinal stem cells, enhances barrier function, and decreases tumor burden in organoid and mouse CRC models. This highlights its protective potential (84).

Conversely, DCA and tauro-β-muricholic acid (T-βMCA) antagonize FXR signaling, promoting Lgr5^+^ stem cell proliferation, DNA damage, and tumorigenesis (89). Reduced FXR expression alters bile acid composition and promotes colonization by *enterotoxigenic Bacteroides fragilis*. This, in turn, enhances secretory IgA production, biofilm formation, and tumor progression (90). Elevated DCA levels can further suppress CD8^+^ T cell effector functions by inhibiting plasma membrane Ca^2+^ ATPase (PMCA) and the Ca^2+^–NFAT2 axis. This suppression correlates with impaired T cell activity in CRC patients (91). High-fat diet (HFD)–induced elevation of BSH activity leads to increased production of DCA and LCA. This, in turn, activates the β-catenin–CCL28 pathway and recruits FOXP3^+^ Tregs, which promotes tumor progression. Notably, this process can be reversed by inhibiting BSH (92).

In addition to tumor promotion, excessive DCA and LCA influence chemotherapy response by downregulating P-glycoprotein, increasing intracellular 5-FU accumulation, and aggravating intestinal toxicity (93). Collectively, FXR-activating bile acid derivatives, such as 3-oxo-LCA, function as tumor suppressors. In contrast, DCA and LCA contribute to epithelial injury, immune suppression, and tumor growth. Therefore, modulating bile acid metabolism, BSH activity, or FXR signaling may represent effective therapeutic strategies for CRC.

### 3.3 Trimethylamine-N-oxide

TMAO is a secondary metabolite generated from microbial metabolism of choline, betaine, and carnitine. Initially linked to cardiovascular diseases such as atherosclerosis and thrombosis (94, 95), TMAO has more recently been implicated in CRC pathogenesis. Elevated circulating TMAO promotes inflammation, oxidative stress, endoplasmic reticulum stress, and insulin resistance—all of which favor tumor progression (96).

In obese CRC patients, reduced abundance of butyrate-producing bacteria correlates with elevated plasma TMAO levels, increased IL-1β expression, and impaired intestinal barrier function (97). Mechanistically, TMAO upregulates IL-6 and multiple chemokines, enhances angiogenesis by inducing VEGFA and CD31, and activates the NLRP3 inflammasome, promoting tumor growth and metastasis (98, 99). TMAO also inhibits the FXR–FGF15 axis while activating Wnt/β-catenin signaling, thereby stimulating tumor cell proliferation, reducing apoptosis, and disrupting epithelial integrity (100). In parallel, TMA and TMAO upregulate SREBF1 and activate the PI3K/AKT pathway, further promoting CRC development (101). Mouse xenograft studies confirm that a high-TMAO diet significantly accelerates tumor growth compared to controls. These findings collectively establish TMAO as a pro-tumorigenic microbial metabolite that alters the inflammatory and metabolic landscape of the tumor microenvironment.

Collectively, these microbial metabolites not only reshape the tumor microenvironment but also directly influence intestinal stem cell behavior and cancer stem cell (CSC) dynamics, thereby contributing to tumor initiation.

## 4 Microbiota and cancer stem cell dynamics in tumor initiation

Accumulating evidence indicates that colorectal tumor initiation is driven by a subpopulation of CSCs, characterized by self-renewal capacity, multipotency, and sustained tumor-propagating potential (102–104). While genetic alterations such as APC loss initiate aberrant Wnt signaling, emerging studies suggest that gut microbiota and their derived signals play a critical role in modulating CSC properties and shaping the tumor-initiating niche (105–107). This microbiota–CSC axis provides a mechanistic bridge linking early tumorigenesis with downstream metabolic and immune remodeling.

### 4.1 Microbiota-driven activation of stemness signaling pathways

Gut microbes can directly influence CSC maintenance by modulating canonical stemness-associated signaling pathways. Among these, Wnt/β-catenin signaling represents a central axis governing intestinal stem cell identity and colorectal tumor initiation. Pathogenic bacteria such as Fusobacterium nucleatum activate β-catenin signaling through FadA-E-cadherin interactions, leading to transcriptional upregulation of stemness-associated genes, including LGR5, CD44, and MYC (18, 108). Similarly, enterotoxigenic Bacteroides fragilis (ETBF) promotes epithelial transformation and stem-like phenotypes via toxin-mediated activation of STAT3 and NF-κB signaling (33, 56). In addition to Wnt signaling, microbial cues have also been implicated in the modulation of Notch and Hedgehog pathways, further reinforcing CSC self-renewal and lineage plasticity. These findings suggest that dysbiotic microbiota actively reprogram epithelial cells toward a stem-like state, thereby lowering the threshold for tumor initiation.

### 4.2 Microbial metabolites in CSC regulation

Beyond direct bacterial-host interactions, microbiota-derived metabolites critically regulate CSC behavior through metabolic and epigenetic mechanisms. SCFAs, particularly butyrate, exhibit context-dependent effects on stemness. In normal intestinal epithelium, butyrate supports differentiation and maintains homeostasis; however, in transformed cells, its accumulation functions as a histone deacetylase (HDAC) inhibitor, leading to suppression of proliferation and induction of differentiation (55). Notably, this dual role extends to CSCs, where butyrate can either restrict stemness through epigenetic repression or, under specific metabolic conditions, support CSC survival (109).

In parallel, secondary bile acids contribute to CSC regulation by reshaping the intestinal niche (91). Tumor-promoting bile acids such as deoxycholic acid (DCA) enhance LGR5^+^ intestinal stem cell proliferation, induce DNA damage, and facilitate malignant transformation, whereas FXR-activating bile acid derivatives exert suppressive effects on stem cell expansion (89). These observations highlight microbial metabolites as key determinants of CSC fate, acting through both chromatin remodeling and niche signaling pathways.

### 4.3 Microbiota modulation of the CSC niche

The maintenance of CSCs is critically dependent on their surrounding microenvironment, or niche, which is strongly influenced by microbiota-driven inflammation and immune signaling (110). Chronic exposure to proinflammatory cytokines such as IL-6, IL-1β, and TNF-α activates STAT3 and NF-κB pathways, promoting CSC self-renewal and survival. In particular, IL-6/STAT3 signaling has been shown to sustain stemness and enhance tumor-initiating capacity (111). In addition, microbiota-induced Th17 responses and IL-17 production further contribute to a pro-tumorigenic niche that supports CSC expansion.

Microbiota-driven immune remodeling also indirectly regulates CSCs by shaping myeloid cell populations and suppressing antitumor immunity (112). The accumulation of myeloid-derived suppressor cells (MDSCs) and M2-like macrophages creates an immunosuppressive microenvironment that facilitates CSC persistence and therapeutic resistance (113). Notably, disruption of epithelial barrier integrity further amplifies this process by allowing microbial components to access the lamina propria, thereby sustaining chronic inflammation and reinforcing CSC-supportive conditions (114).

Collectively, these findings indicate that the gut microbiota not only influences tumor progression through immune and metabolic pathways but also directly participates in early colorectal tumorigenesis by regulating CSC properties and their niche. It has also been proposed that a subset of CSCs may possess intrinsic metastatic potential at early stages of tumorigenesis, further underscoring the importance of microbiota–CSC interactions in shaping disease trajectory.

## 5 Microbiota-mediated immune and signaling mechanisms in colorectal cancer

The gut microbiota plays a crucial role in shaping the intestinal immune microenvironment and orchestrating signaling cascades that contribute to colorectal carcinogenesis. Dysbiosis triggers chronic mucosal inflammation through pattern recognition receptors like TLRs and NLRs. This process leads to the activation of NF-κB and STAT3, resulting in the production of proinflammatory cytokines such as IL-6, IL-17, and TNF-α. These cytokines, in turn, promote epithelial proliferation, angiogenesis, and DNA damage, creating an environment conducive to tumor development.

### 5.1 Induction of inflammation

Inflammation is a hallmark of cancer and an established risk factor for CRC (115). Gut dysbiosis is closely linked to inflammation in the GI tract and plays a significant role in the development of colitis-associated CRC. It can promote chronic inflammation through various mechanisms. Disruption of microbial homeostasis increases the abundance of pathogenic or proinflammatory taxa, such as *F. nucleatum*, ETBF, and *Escherichia coli* carrying the pks genomic island, which produce virulence factors and toxins that activate host immune pathways (19, 22, 36). These bacteria stimulate epithelial and immune cells via pattern recognition receptors, including Toll-like receptors (TLRs) and NOD-like receptors (NLRs), leading to activation of NF-κB, MAPK, and STAT3 signaling (52, 116). The upregulation of cytokines such as IL-1β, IL-6, TNF-α, and IL-17 leads to a proinflammatory microenvironment that enhances epithelial proliferation and facilitates DNA damage, contributing to tumor initiation. Furthermore, microbial metabolites and structural components—such as lipopolysaccharide (LPS), flagellin, and peptidoglycan—intensify mucosal inflammation by recruiting myeloid cells and sustaining the release of cytokines (117). Persistent exposure to these inflammatory cues drives aberrant epithelial regeneration and fosters a transition from colitis to colorectal neoplasia. Thus, microbiota-induced inflammation represents a critical mechanistic link between gut dysbiosis and colorectal tumorigenesis.

### 5.2 Suppression of inflammation

In contrast, several commensal bacteria exert anti-inflammatory and protective effects that maintain intestinal homeostasis and prevent tumor-promoting inflammation. Beneficial taxa such as *F. prausnitzii*, *Akkermansia muciniphila*, and butyrate-producing *Clostridium* species contribute to epithelial barrier integrity and modulate immune responses (10, 60, 118). Their metabolites, especially SCFAs such as butyrate and propionate, suppress the production of proinflammatory cytokines. They achieve this by inhibiting NF-κB activation in macrophages and epithelial cells. Additionally, these metabolites promote the differentiation of Tregs through the epigenetic modulation of the FOXP3 locus (72, 78).

Moreover, microbial-derived indole derivatives, secondary bile acids, and tryptophan metabolites act as ligands for the aryl hydrocarbon receptor (AhR) and G-protein–coupled receptors (GPCRs), thereby enhancing IL-22 production by innate lymphoid cells and strengthening mucosal repair (119–121). These anti-inflammatory pathways work together to limit chronic inflammation and genotoxic stress, thereby reducing the risk of colitis-associated colorectal carcinogenesis. Consequently, it is essential to maintain a balanced gut microbiota composition and ensure the production of anti-inflammatory metabolites to suppress tumor-promoting inflammation and preserve intestinal immune equilibrium.

### 5.3 Promoting immune evasion

Beyond driving inflammation, dysregulated gut microbiota also contributes to CRC progression by promoting immune evasion. Certain pathogenic bacteria, such as *F. nucleatum*, *Bacteroides fragilis*, and *Escherichia coli*, have evolved strategies to suppress anti-tumor immune responses within the tumor microenvironment. *F. nucleatum*, expresses the outer membrane adhesin Fap2, which binds to the inhibitory receptor TIGIT on natural killer (NK) cells and CD8^+^ cytotoxic T cells. This binding hinders their cytolytic activity, facilitating tumor immune escape (20). In addition, *F. nucleatum* can recruit MDSCs and TAMs via TLR4–NF-κB signaling, amplifying local immunosuppression and supporting tumor growth (19).

Similarly, ETBF induces Th17-polarized immune responses and secretes BFT, which activates STAT3 and β-catenin signaling, creating an immunosuppressive and pro-tumorigenic environment (32). Other microbial components, such as LPS and peptidoglycan, can chronically stimulate innate receptors (TLRs, NOD-like receptors), leading to immune exhaustion and the expansion of IL-10–producing Treg (120). These processes diminish antigen presentation, impair cytotoxic lymphocyte activation, and weaken immune surveillance.

Importantly, microbial metabolites also directly regulate immune checkpoint pathways, particularly the PD-1/PD-L1 axis. SCFAs such as butyrate and propionate can enhance antitumor immunity by increasing CD8^+^ T-cell effector function, promoting memory differentiation, and modulating histone acetylation at immune-regulatory loci, thereby improving responsiveness to PD-1 blockade (74, 79, 80). In contrast, depletion of beneficial SCFAs during dysbiosis is associated with reduced T-cell fitness and impaired checkpoint immunotherapy efficacy.

Beyond SCFAs, other microbiota-derived metabolites have emerged as critical modulators of checkpoint responses. Inosine, produced by commensal bacteria such as Bifidobacterium species, can activate A2A receptor signaling in T cells under appropriate co-stimulatory conditions, enhancing Th1 differentiation and potentiating anti-PD-1 immunotherapy (122). Likewise, tryptophan-derived indole metabolites act through the aryl hydrocarbon receptor (AhR) to regulate dendritic cell maturation, barrier immunity, and T-cell polarization, thereby influencing PD-L1 expression and the balance between immune activation and tolerance (122, 123). Conversely, excessive kynurenine pathway activity may promote T-cell dysfunction and checkpoint resistance.

Furthermore, dysbiosis-induced metabolic alterations—such as decreased butyrate and increased polyamine and TMAO production—can epigenetically reprogram immune cells toward a tolerogenic phenotype, further promoting immune escape (95). These mechanisms collectively demonstrate that an imbalanced microbiota triggers inflammation and allows tumor cells to evade immune detection. This underscores the dual role of the microbiota in both initiating and sustaining colorectal carcinogenesis.

### 5.4 Microbiota-driven modulation of oncogenic signaling pathways

Gut microbiota can directly influence oncogenic signaling pathways in the intestinal epithelium, establishing a connection between microbial dysbiosis and colorectal tumorigenesis. Various bacteria and their metabolites activate proliferative and anti-apoptotic signaling cascades, including Wnt/β-catenin, NF-κB, STAT3, and PI3K/AKT (**Table 2**). Together, these pathways promote epithelial transformation, tumor growth, and metastasis (2).

**Table 2:** Regulation of key oncogenic signaling pathways by gut microbiota and their metabolites.

Category	Role	Key effector molecule	Major pathways regulated	Biological effects
*F. nucleatum*	Driver	FadA	Wnt/β-catenin	Binds E-cadherin → β-catenin nuclear translocation → activation of MYC, CCND1 → enhanced proliferation & tumor progression (18)
*ETBF*	Driver	BFT	STAT3, NF-κB	E-cadherin cleavage → pro-survival signaling, chronic inflammation, epithelial transformation (32)
*CoPEC*	Driver	Colibactin	DNA damage response; p53-associated pathways	DNA double-strand breaks → genomic instability → activation of oncogenic transcription programs (26, 27)
Microbial PAMPs	Promoter	LPS/flagellin/CpG DNA	TLR2/4/9 → MyD88–NF-κB	COX-2, IL-6, TNF-α upregulation; chronic inflammation–associated carcinogenesis (52)
Secondary bile acids	Promoter	DCA	EGFR–MAPK; Wnt/β-catenin	ROS generation, increased proliferation, apoptosis inhibition (53, 54)
Loss of SCFAs	Loss of protective factor	Butyrate	Histone acetylation; tumor-suppressive gene regulation	Reduced HDAC inhibition → decreased tumor-suppressive transcription → favors carcinogenesis (55)

Driver/Promoter indicates factors that activate pathways; Loss of protective factor indicates absence of inhibitory signals. Arrows (→) denote sequential molecular events.

Among these, *F. nucleatum* has been shown to activate the Wnt/β-catenin pathway through its virulence factor FadA, which binds to E-cadherin on epithelial cells, triggering β-catenin nuclear translocation and transcription of oncogenic targets such as MYC and CCND1 (18). Similarly, ETBF secretes B. fragilis toxin (BFT), which induces E-cadherin cleavage and activates STAT3 and NF-κB signaling (32). Persistent activation of these pathways enhances the expression of pro-survival and proliferative genes while supporting a cytokine milieu that favors chronic inflammation and tumor progression.

**Figure 4:**
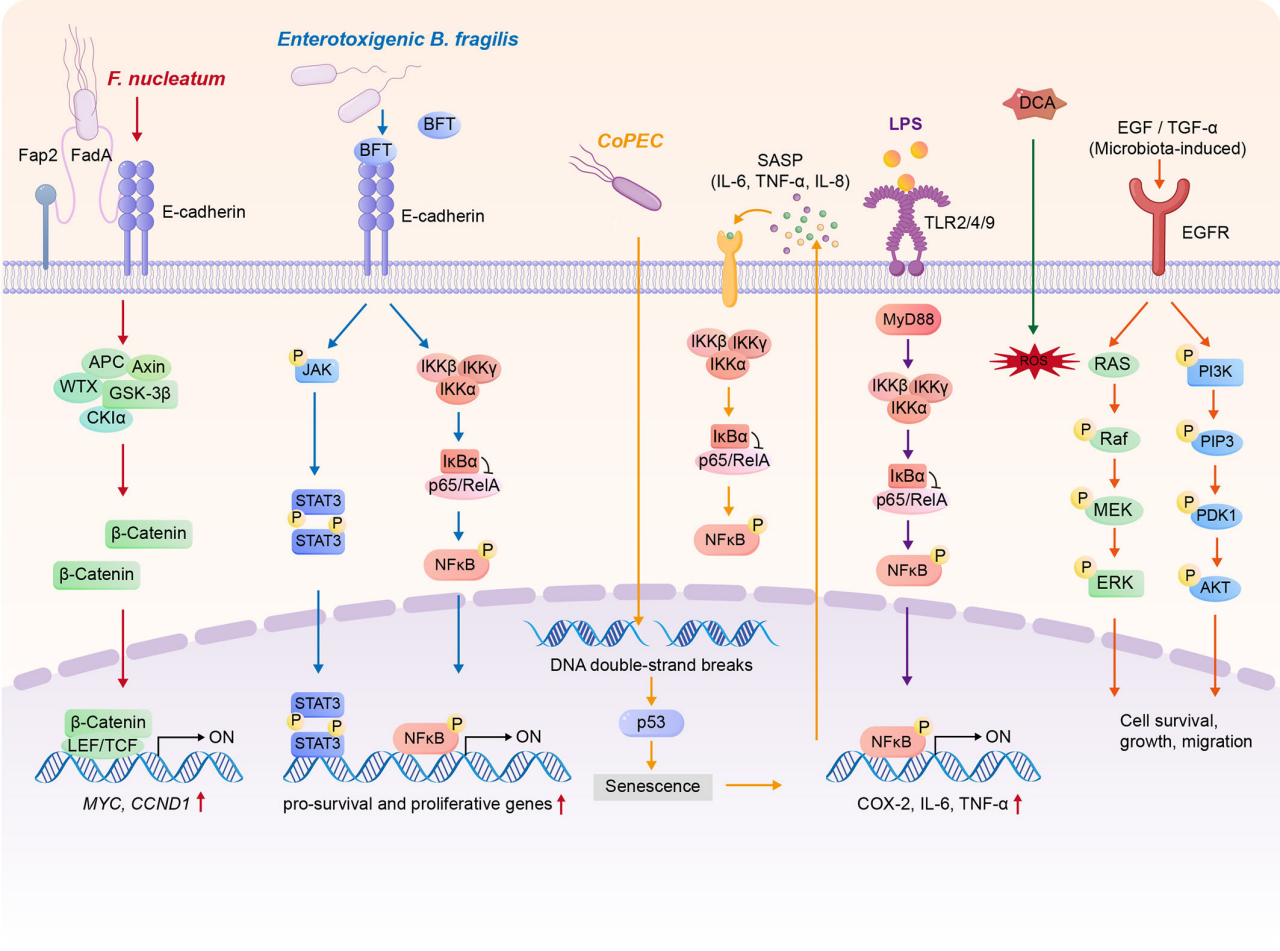
Oncogenic microorganisms (such as F. nucleatum, ETBF) and their metabolites (such as DCA) activate core oncogenic signaling pathways like Wnt/β-catenin, NF-κB, and STAT3 through specific virulence factors or signaling molecules. Simultaneously, genotoxic bacteria (like CoPEC) can directly induce DNA damage. Conversely, the depletion of protective metabolites (like butyrate) weakens their inhibitory function on these pathways. The synergistic action of these mechanisms ultimately collectively promotes carcinogenic phenotypes such as cell proliferation, inflammation, stem cell characteristics, and genomic instability.

Other bacteria, including CoPEC strains, can induce DNA double-strand breaks and activate the DNA damage response, leading to genomic instability and sustained activation of oncogenic transcription factors (26, 27). Bacterial LPS, flagellin, and CpG DNA engage Toll-like receptors (TLR2, TLR4, TLR9), activating MyD88-dependent NF-κB signaling, which promotes the expression of COX-2, IL-6, and TNF-α—key mediators of inflammation-associated carcinogenesis (52). In addition to microbial components, metabolites function as signaling molecules that modulate oncogenic cascades. For instance, secondary bile acids like DCA can induce the production of reactive oxygen species (ROS) and activate the EGFR–MAPK and Wnt/β-catenin signaling pathways. This activation enhances cellular proliferation while suppressing apoptosis (53, 54). Conversely, the loss of SCFAs, particularly butyrate, leads to impaired histone acetylation and reduced tumor-suppressive gene expression, further tipping the balance toward tumorigenic signaling (55). These findings collectively indicate that the microbiota plays a crucial role in influencing immune regulation and altering epithelial signaling networks in a way that promotes carcinogenesis (**Figure 4**). Therefore, targeting these microbial–epithelial signaling axes could serve as a promising therapeutic strategy for both the prevention and treatment of colorectal cancer.

### 5.5 Multi-omics and spatial profiling reveal heterogeneity of microbiota–immune interactions in CRC

Recent advances in multi-omics technologies have substantially expanded our understanding of the complex and heterogeneous interactions between the gut microbiota, tumor cells, and host immunity in CRC. Conventional single-layer analyzes often fail to capture the dynamic crosstalk among microbial composition, metabolic function, and immune signaling. In contrast, integrated approaches combining metagenomics, metabolomics, transcriptomics, and spatially resolved profiling now provide a more comprehensive framework for defining patient-specific tumor ecosystems (124–126).

Metagenomic sequencing has shown that CRC patients harbor highly diverse microbial communities, with enrichment of taxa such as Fusobacterium nucleatum, enterotoxigenic Bacteroides fragilis, Escherichia coli, and oral pathobionts in specific patient subsets. However, taxonomic abundance alone does not fully explain disease behavior. Functional metagenomics further demonstrates marked interpatient variation in pathways related to amino acid metabolism, bile acid transformation, polyamine biosynthesis, lipopolysaccharide production, and short-chain fatty acid synthesis, indicating that microbiome functionality may be more informative than composition alone (126).

When integrated with metabolomics, these datasets reveal distinct metabolic phenotypes associated with immune states. For example, tumors enriched in butyrate-producing bacteria often exhibit enhanced epithelial differentiation and increased cytotoxic T-cell infiltration, whereas microbiota associated with secondary bile acids, TMAO, lactate, or polyamines tend to correlate with immunosuppressive microenvironments characterized by Tregs, MDSCs, and exhausted T cells. Such findings support the concept that microbial metabolites serve as functional mediators linking compositional dysbiosis to immune remodeling (127).

Transcriptomic analyzes further uncover host-response heterogeneity across CRC subtypes (128). Microbiota-high tumors frequently display activation of NF-κB, IL-6/STAT3, Wnt/β-catenin, interferon, and checkpoint-related pathways, accompanied by elevated expression of PD-L1, CXCL chemokines, and inflammatory cytokines. In contrast, other tumors exhibit immune-desert or stromal-excluded phenotypes despite similar clinicopathologic staging, suggesting that microbiota-associated immune programs may contribute to differences in prognosis and therapeutic responsiveness.

More recently, spatial transcriptomics and multiplex imaging have enabled *in situ* mapping of microbial–immune interactions within tumor tissues. These approaches demonstrate that bacteria are not uniformly distributed but localize to specific niches, including invasive fronts, hypoxic glands, mucus-rich regions, and immune cell–dense stromal compartments (129, 130). Proximity of F. nucleatum or other intratumoral bacteria to macrophages, neutrophils, and exhausted CD8^+^ T cells has been associated with localized inflammatory signaling and immune suppression (79). Spatial analyzes also reveal substantial intratumoral heterogeneity, where immune-active and immune-suppressed regions may coexist within the same lesion (131).

Collectively, multi-omics and spatial studies indicate that CRC is not defined by a single microbiome signature, but by multiple microbiota–immune ecological states with significant interpatient and intratumoral heterogeneity. These findings provide a strong rationale for precision oncology strategies that incorporate microbial biomarkers, metabolic signatures, and spatial immune architecture to improve patient stratification and guide personalized interventions.

## 6 Clinical implications: microbiome and metabolite-based strategies for early diagnosis, prognosis, and treatment prediction in CRC

Accumulating evidence indicates a strong translational potential of gut microbiota and their metabolites in the clinical management of CRC (2, 61). Microbial dysbiosis not only precedes and accompanies malignant transformation, but also actively influences tumor biology, host immunity, and therapeutic responsiveness. These features position the microbiome as a multidimensional resource for risk assessment, non-invasive diagnosis, prognostic stratification, treatment prediction, and therapeutic intervention. Importantly, however, current clinical application requires careful distinction between associative biomarkers identified in observational studies and causally validated microbial or metabolic effectors supported by mechanistic evidence.

### 6.1 Early detection and non-invasive screening

In early detection, fecal microbial signatures can identify early-stage CRC and advanced adenomas with high sensitivity, particularly for proximal colon lesions that are frequently missed by standard screening approaches. The enrichment of oncogenic bacteria such as *F. nucleatum*, *Peptostreptococcus stomatis*, *Parvimonas micra*, and enterotoxigenic *Bacteroides fragilis*, along with the depletion of SCFA-producing commensals, has been validated as a robust diagnostic panel, which substantially enhances the sensitivity of fecal immunochemical tests (15, 16, 39, 57, 132–135).

Beyond taxonomic markers, integration of microbial functional genes (e.g., pks island), metagenomic pathway signatures, and stool metabolite profiles—including reduced butyrate and elevated TMAO or secondary bile acids—may further enhance personalized and non-invasive CRC screening performance (22, 96, 136). Recent cross-cohort studies, including Piccinno G et al. (Nat Med, 2025) (61), suggest that a subset of microbiome-derived markers is reproducible across geographically distinct populations, supporting future clinical deployment of robust microbial screening models.

### 6.2 Prognostic stratification and disease monitoring

Microbial and metabolite features can also be used to predict the prognosis of disease (137). Specific microbial colonization patterns are strongly associated with tumor stage, molecular subtype, and patient outcomes. For example, intratumoral *F. nucleatum* enrichment correlates with the CMS4 subtype, elevated epithelial–mesenchymal transition signatures, metastatic potential, and reduced survival (138), while dysregulation of bile acid metabolism and increased circulating TMAO levels predict higher recurrence and poorer response to standard therapies (20, 96).

Future prognostic frameworks should move beyond single-marker associations toward integrated patient stratification based on both microbiome composition and immune contexture. Predictive models incorporating F. nucleatum abundance, circulating TMAO levels, butyrate-producing capacity, MSI status, PD-L1 expression, and CD8^+^ T-cell infiltration may provide more accurate estimates of recurrence, survival probability, and treatment responsiveness than conventional clinicopathologic parameters alone.

In addition, longitudinal microbiome monitoring during therapy may enable real-time assessment of disease progression, recurrence, and emerging resistance.

### 6.3 Prediction of therapeutic response

Besides being biomarkers, the microbiome can influence therapeutic efficacy (139). Intratumoral pathogens may induce chemotherapy resistance through autophagy activation, while SCFA-producing bacteria enhance cytotoxic T cell activity and sensitivity to immune checkpoint blockade (70, 71, 99). Several studies have shown that microbiome profiles can alter response to immunotherapy response, providing a basis for patient stratification and microbiota-informed treatment decision-making (140).

Mechanistically supported examples include targeting F. nucleatum, restoring butyrate-producing microbial networks, and modulating bile acid signaling pathways (73, 141). However, many probiotic formulations and composite microbiome signatures currently lack standardized composition, reproducible efficacy, and prospective multicenter validation.

### 6.4 Microbiota-targeted therapeutic strategies and ongoing clinical trials

Interventions designed to remodel the intestinal ecosystem are being actively explored as adjunctive CRC therapies. These include dietary fiber enrichment, prebiotics, probiotics, engineered bacterial consortia, postbiotic/metabolite supplementation, bile acid enzyme inhibitors, antibiotics targeting pathobionts, and fecal microbiota transplantation (FMT) (142, 143). Such approaches aim to restore eubiosis, reinforce barrier integrity, reduce carcinogenic metabolites, and enhance anti-tumor immunity.

Importantly, several microbiota-directed strategies have entered clinical evaluation. Ongoing trials are assessing FMT combined with PD-1 blockade rechallenge in resistant metastatic dMMR/MSI-H CRC, as well as FMT plus first-line systemic therapy in unresectable CRC (**Table 3**). These studies may clarify feasibility, donor selection principles, engraftment efficiency, safety, and whether microbiome modulation can generate durable therapeutic benefit.

**Table 3:** Selected ongoing clinical trials of microbiota-based interventions relevant to CRC.

NCT number	Intervention	Population	Phase	Status
NCT04729322	FMT + re-introduction of pembrolizumab/nivolumab	Metastatic dMMR colorectal adenocarcinoma with prior anti-PD-1 non-response	Early phase I (pilot)	Ongoing
NCT07509398	FMT + first-line standard therapy	Initially unresectable advanced metastatic colorectal cancer (mCRC)	Phase II/III	Not yet recruiting
NCT03819296*	Microbiome profiling ± FMT for immune checkpoint inhibitor-related GI toxicity	Mixed solid tumors (including GI malignancies)	Phase I	Active

*Not CRC-specific, but clinically relevant to microbiota-directed immunotherapy management.

### 6.5 Current limitations and future directions

Despite rapid progress, several barriers must be addressed before microbiome-based strategies can be routinely integrated into CRC care. First, technical standardization remains a major challenge, including harmonized procedures for stool/tissue sampling, storage conditions, sequencing platforms, bioinformatic pipelines, and metabolite quantification. Variability introduced by diet, antibiotics, bowel preparation, geography, and ancestry can substantially affect reproducibility across cohorts.

Second, causality must be clearly distinguished from correlation. While F. nucleatum, butyrate, and DCA have substantial mechanistic evidence linking them to CRC progression or immune modulation, many other candidate taxa currently function only as associative biomarkers. Establishing clinically actionable targets will require gnotobiotic models, organoid systems, intervention trials, and longitudinal human studies.

Third, patient stratification will be essential. Future precision frameworks should integrate microbiome features with metabolomic, genomic, radiologic, and immunologic parameters, potentially supported by artificial intelligence–based modeling. Such multidimensional tools may outperform conventional staging systems for screening, prognosis, and therapy selection.

Overall, microbiome- and metabolite-based strategies represent a promising frontier in CRC precision medicine. Their successful clinical translation will depend on standardized methodologies, multicenter validation, biologically informed patient stratification, and carefully designed interventional trials.

## 7 Conclusions and future directions

The gut microbiota has emerged as a central player in the pathogenesis of colorectal cancer, bridging metabolic reprogramming, immune dysregulation, and epithelial transformation. Accumulating evidence highlights that microbial metabolites are not just by-products of bacterial activity; rather, they are powerful bioactive mediators that regulate host signaling networks involved in inflammation, DNA integrity, and tumor immunity. The dynamic interplay among SCFAs, secondary bile acids, and tryptophan metabolites exemplifies how microbial metabolism can either sustain intestinal homeostasis or promote malignant transformation, depending on contextual cues within the tumor microenvironment.

Despite significant progress, several key challenges persist. The causal hierarchy connecting specific microbial taxa, metabolites, and host signaling pathways in CRC remains incompletely understood. Interindividual microbiome heterogeneity, metabolic redundancy, and the impacts of diet and genetics complicate the interpretation of mechanisms and the translation of therapies. Future research should focus on integrating multi-omics profiling, spatial microbiome mapping, and advanced organ Additionally, interindividual oid or immune co-culture models to unravel these complex networks with both temporal and spatial precision.

Ultimately, the microbiota–metabolite–immune axis represents a promising frontier for CRC prevention and therapy. Personalized strategies—combining dietary modulation, probiotics, prebiotics, postbiotics, or FMT—with conventional treatments may offer synergistic benefits. A deeper understanding of microbial metabolism and immune crosstalk will pave the way for microbiome-based precision medicine. This advancement will transform the management of colorectal cancer from generalized interventions to individualized microbial modulation.
